# Effect of Caudal Epidural Steroid Injection on Transforaminal Epidural Steroid Injection and Dorsal Root Ganglion Pulsed Radiofrequency in Recurrent Lumbar Disc Herniation

**DOI:** 10.3390/jcm13247821

**Published:** 2024-12-21

**Authors:** Gülçin. Gazioğlu Türkyılmaz, Şebnem. Rumeli, Mesut. Bakır, Suna. Aşkın Turan

**Affiliations:** 1 Pain Clinic, Bursa City Hospital, Bursa 16110, Turkey; 2 Division of Pain Medicine, Department of Anesthesiology and Reanimation, Mersin University Faculty of Medicine, Mersin 33240, Turkey; sebnemrumeli66@gmail.com (Ş.R.); mesutbakir@mersin.edu.tr (M.B.); 3 Pain Clinic, Mersin City Education and Research Hospital, Mersin 33343, Turkey; sunaaskin1@gmail.com

**Keywords:** back pain, intervertebral disc, recurrence, pulsed radiofrequency treatment, steroids, pain measurement

## Abstract

**Background/Objectives:** Recurrent lumbar disc herniation (RLDH) refers to a lumbar disc herniation (LDH) that recurs at the same level, location, and side following surgical repair. This study aimed to evaluate the efficacy of transforaminal epidural steroid injection (TESI) and dorsal root ganglion pulsed radiofrequency (DRG PRF) therapy with and without caudal epidural steroid injection (CESI) for the treatment of lumbar radicular pain (LRP) associated with RLDH. **Methods:** This retrospective cohort study included 57 patients treated for RLDH in a hospital pain clinic between September 2022 and February 2024. A total of 27 patients received TESI and DRG PRF therapy (Group 1) and 30 patients received TESI, DRG PRF, and CESI therapy (Group 2). We evaluated patient age, sex, symptom duration, pain medication use, number of prior LDH operations, presence of stabilization on magnetic resonance imaging (MRI), intervention received, lumbar level and side of the intervention, and Numeric Rating Scale (NRS) pain scores before and at 1, 3, and 6 months post-procedure. Treatment success was defined as an NRS score at least 50% or 4 points lower than the pre-procedure score at post-procedure 6 months. **Results:** There was no significant difference in NRS scores between the groups during the 6-month follow-up period. Moreover, NRS scores did not differ based on the presence of stabilization on MRI or the use of pain medication (*p* > 0.05). **Conclusions:** TESI and DRG PRF therapy were effective in the treatment of LRP associated with RLDH over a 6-month follow-up period, and adding CESI did not increase treatment success.

## 1. Introduction

Recurrent lumbar disc herniation (RLDH) refers to a lumbar disc herniation (LDH) that recurs at the same level, location, and side after surgical repair. The recurrence rate of LDH is reported to be 5% to 15%. RLDH is diagnosed using magnetic resonance imaging (MRI) when lumbar radicular pain (LRP) reoccurs in the same region after at least 6 months of being pain-free postoperatively [1,2].

Chronic pain has emotional and attentional components and can significantly impact overall well-being. In addition to being a predominant clinical presentation, chronic pain is a major contributor to disability and imposes substantial socioeconomic burdens. The management of symptomatic RLDH focuses on pain relief and follows a structured, stepwise approach that should be individualized to each patient and implemented using a multimodal strategy. Clinical guidelines recommend starting with conservative treatment modalities. When conservative approaches such as analgesic medications and physical therapy fail to effectively control symptoms, interventional procedures should be considered.

The optimal treatment option for these patients remains controversial. Repeated invasive interventions are associated with complications such as dural injury, nerve injury, and lower success rates in spine surgery [3]. The efficacy of transforaminal epidural steroid injection (TESI) and caudal epidural steroid injection (CESI) alone and in combination have been demonstrated in RLDH [4,5,6]. There are also studies showing that dorsal root ganglion pulsed radiofrequency (DRG PRF) therapy in combination with TESI in the treatment of LRP provides more effective pain control for 3 months compared to TESI alone [7,8]. However, we found no studies in the literature evaluating the effectiveness of DRG PRF therapy for LRP in RLDH.

This study aimed to evaluate whether the addition of CESI to TESI and DRG PRF improves treatment success in patients with RLDH.

## 2. Materials and Methods

### 2.1. Ethical Approval and Patient Consent

The study was approved by the Clinical Research Ethics Committee of Bursa City Hospital (decision number 2024-2/3, date: 21 February 2024) and followed the Declaration of Helsinki. All study participants gave written and verbal informed consent. The Strengthening the Reporting of Observational Studies in Epidemiology (STROBE) guidelines were followed in writing the manuscript.

### 2.2. Study Design and Participants

This retrospective cohort study included patients who underwent LDH surgery more than 6 months ago and were treated in the pain outpatient clinic between 1 September 2022 and 29 February 2024. The patients presented with neuropathic pain that radiated to the lower back and leg on the operated side for more than 3 months and the absence of surgical indications such as loss of dorsal or plantar flexion, drop foot, or cauda equina syndrome on initial examination. All patients reported severe pain (Numeric Rating Scale [NRS] score greater than 4 at their initial visit), had previously undergone conservative treatments such as medical and physical therapy with no improvement in pain, were diagnosed with RLDH by MRI, had no sequestrated disc on MRI, had no systemic infection or local infection at the intervention site, and had complete data from the follow-up period. Exclusion criteria were having undergone LDH surgery less than 6 months ago; not being treated in the pain outpatient clinic between 1 September 2022 and 29 February 2024; presenting with neuropathic pain radiating to the lower back and leg on the unoperated side; having an NRS score less than 4 at their first visit; having pain for less than 3 months; having not previously undergone any conservative treatments such as medical or physical therapy; having any systemic infection or local infection at the intervention site; having surgical indications such as loss of dorsal or plantar flexion, drop foot, or cauda equina syndrome on initial examination; having sequestered disc on MRI; and having missing data.

RLDH patients who underwent DRG PRF and TESI with fluoroscopic guidance were included in Group 1, while those who underwent DRG PRF and TESI followed by CESI in the same session were included in Group 2.

### 2.3. Intervention

The same pain physician (G.G.T.) evaluated all patients in the study and performed all DRG PRF, TESI, and CESI procedures under sterile conditions with standard monitoring and mild sedation (1 or 2 mg midazolam).

#### 2.3.1. DRG PRF and TESI

The procedure was performed as described previously [9]. Briefly, in prone position, patients received 1 mL 2% lidocaine by skin infiltration. Under ipsilateral oblique (25–30°) tunnel view C-arm fluoroscopic guidance, a 10 cm 22-gauge radiofrequency needle with a 10 mm active tip (TOP, Tokyo, Japan) was advanced medially as close as possible to the target DRG without passing the midline of the intervertebral foramen in the lateral view or the midline of the pedicle column in the anteroposterior view. Using a radiofrequency generator (TOP-TLG 10 STP, TOP, Tokyo, Japan), appropriate impedance (<400 ohms) and sensory/motor responses were ensured [7,8], then the PRF was applied for 240 s at 45 V and 42 °C.

**Figure 1:**
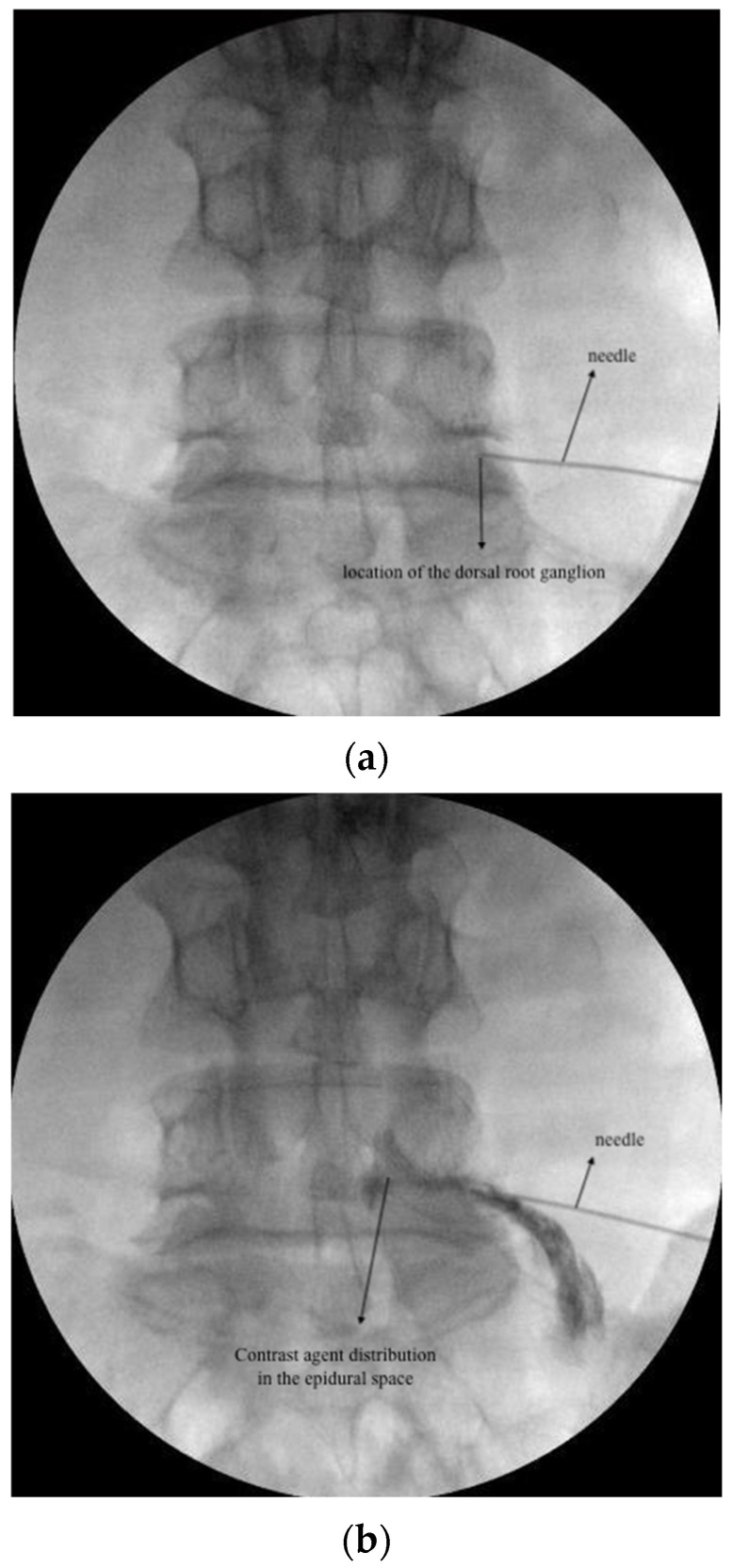

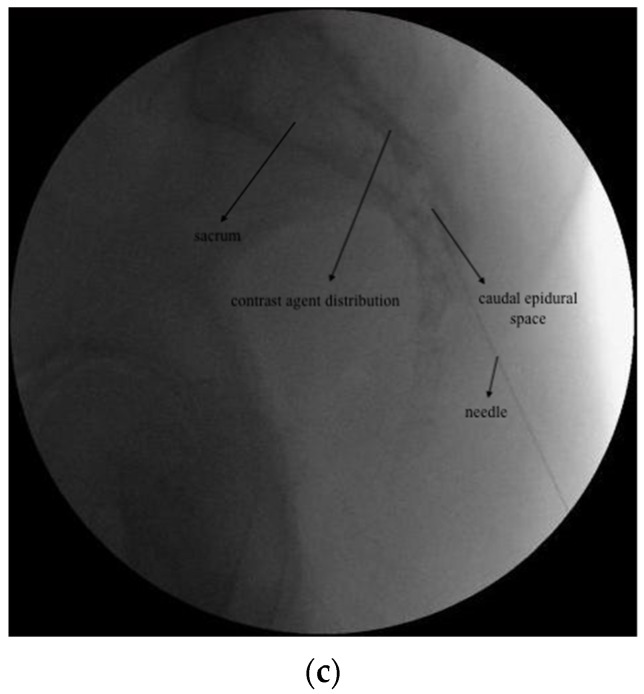
(**a**): DRG PRF fluoroscopic image; (**b**): TESI fluoroscopic image; (**c**) CESI fluoroscopic image.

For TESI, the needle tip was retracted 2–3 mm to realign in the safe triangle following DRG PRF. Epidural spread was verified using contrast medium (Omnipaque 300, GE Healthcare, Dublin, Ireland). A total of 5 mL (4 mg/1 mL dexamethasone and 4 mL 0.9% NaCl) was injected at each target level (Figure 1a,b).

#### 2.3.2. CESI

Under fluoroscopic guidance, the caudal epidural space was visualized in the lateral view. Following skin infiltration with 1 mL 2% lidocaine, a 22-gauge spinal needle was advanced to the caudal epidural space. After visualizing appropriate contrast distribution, a total of 10 mL (40 mg/1 mL methylprednisolone acetate and 9 mL 0.9% NaCl) was injected (Figure 1c).

### 2.4. Outcome Measures and Follow-Up

We retrospectively reviewed the patients’ medical records in the pain department and hospital electronic health system to obtain the following data: age, sex, duration of pain, pain medications used at their initial visit, number of prior LDH operations, patients with and without stabilization on MRI, which intervention was performed (DRG PRF+TESI or DRG PRF+TESI+CESI), the lumbar level and side of the intervention, and NRS values before the procedure and at 1 and 3 months after the procedure. “Stabilization on MRI” was defined as the presence of standard instrumented fusion devices with pedicle screws used to reduce pain associated with anatomic structures by relieving pressure from degenerated discs and facets, as well as stiff rods commonly used in dynamic stabilization devices. The medications used by the patients were not changed during follow-up. We also contacted patients at 6 months after the procedure to assess their NRS levels.

Patients whose NRS score decreased by at least 50% at post-procedure 1 and 3 months compared to their pre-procedure score were regarded as having benefited from treatment. The primary outcome of this study was treatment success, defined as a 50% reduction or a decrease of 4 points in the NRS pain score at 6 months post-procedure compared to the pre-procedure score.

### 2.5. Statistical Analysis

#### 2.5.1. Sample Size Calculation

To determine the required sample size for our investigation, we conducted an a priori power analysis using a study by Evran et al. [4] as a reference. The authors reported 6-month visual analog scale (VAS) leg pain values as 6.19 ± 1.06 for the TESI group (*n* = 32) and 4.23 ± 1.11 for the TESI+CESI group (*n* = 39). Using these values, the effect size was determined as d = 1.81 and accepted as d = 0.80, which was the upper limit of the large effect size [10]. As a result of the a priori power analysis using this effect size value (d = 0.80), type I error of 5%, and target power level of 80%, we determined that a total of 57 individuals should be included in the study to account for possible losses. Analyses were performed using G*Power 3 [11].

#### 2.5.2. Data Analysis

Continuous variables were assessed for normal distribution using the Shapiro–Wilk test. Mean and standard deviation were provided for data that showed a normal distribution, while median, minimum, and maximum values were given for non-normally distributed data. The Mann–Whitney U test or independent-samples *t*-test was used to compare the groups depending on the normality of the data. Categorical variables were expressed as number and percentage. The Pearson chi-square test, Fisher’s exact chi-square test, and Fisher–Freeman–Halton test were used to compare categorical variables between groups. For statistical analyses, SPSS (IBM Corp. Released 2023. IBM SPSS Statistics for Windows, Version 29.0.2.0 Armonk, NY, USA: IBM Corp.) program was used and *p* < 0.05 was considered statistically significant.

## 3. Results

**Figure 2:**
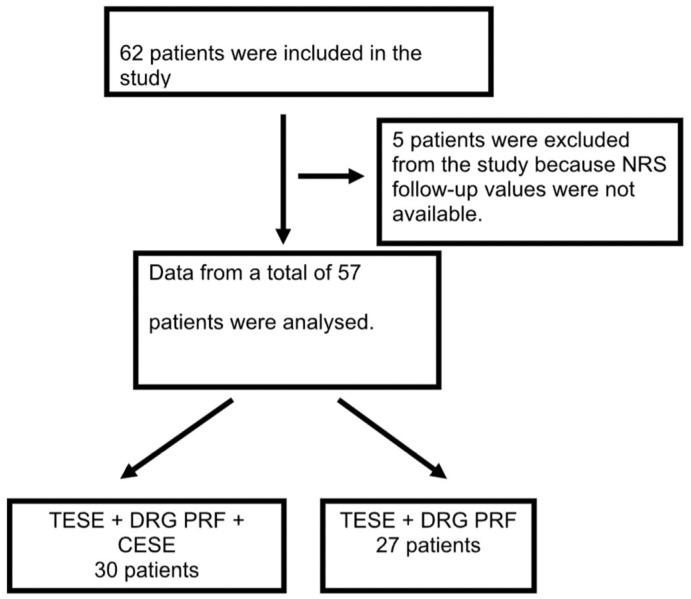
Patient flow chart.

During the 17-month study period, a total of 62 patients underwent either DRG PRF and TESI or DRG PRF, TESI, and CESI treatments for RLDH; 5 of these patients were excluded from the study because follow-up NRS scores were not available. Therefore, data pertaining to a total of 57 patients were examined (30 patients in the DRG PRF+TESI+CESI group and 27 in the DRG PRF+TESI group) (Figure 2).

The patients had a mean age of 57.7 ± 13.0 years and 75.4% (*n* = 43) were female. Injections were most frequently applied on the left side (61.4%) and at the L4–5 and L5-S1s (64.9%). Thirty-one (54.4%) of the patients had one previous LDH surgery. There was no stabilization on MRI in 52.6% of the patients (*n* = 30). The demographic and clinical features of the study group and a comparison of their mean NRS scores are presented in Table 1.

In the DRG PRF+TESI group, the proportion of patients who benefited from treatment at 1 and 3 months was 77.7% (21/27), and the rate of treatment success at 6 months after the procedure was 74% (20/27). In the DRG PRF+TESI+CESI group, 80% (24/30) of the patients benefited from treatment at 1 month and 76.6% (23/30) at 3 months. The rate of treatment success at 6 months was 70% (21/30).

Based on the demographic and clinical characteristics of the patients included in the study, no significant difference was observed between the groups (Table 1, *p* > 0.05). There was no significant difference in NRS scores between the groups during the 6-month follow-up period (*p* > 0.05).

The patients in each group with treatment success at 6 months had similar demographic and clinical characteristics (Table 2, *p* > 0.05).

Table 3 presents a comparison of the patients’ mean NRS values based on the MRI findings related to stabilization. It was determined that there was no difference between the groups according to NRS scores (*p* > 0.05). Stabilization was observed in the MRI of all patients who benefited in the first month and whose pain increased thereafter in both groups.

There was no difference between patients with and without stabilization on MRI in terms of sex, number of previous LDH surgeries, the level and side of the intervention, or use of pain medication (*p* > 0.05).

There was no significant difference in age, sex, number of procedures, presence of stabilization on MRI, and level or side of the procedure between the groups of patients with and without medical pain treatment (*p* > 0.05). The NRS values of patients who did and did not use pain medication are compared in Table 4. There was no difference in NRS scores between the groups (*p* > 0.05).

## 4. Discussion

This is the first study to evaluate the effectiveness of combined DRG PRF and TESI with and without adjuvant CESI for RLDH. At the end of the 6-month follow-up period, both groups had a treatment success rate higher than 70%. The lack of a significant difference between the groups suggests that DRG PRF is the main factor in treatment success.

There are many studies in the literature investigating risk factors for RLDH. Suk et al. [2] found that young age and male sex were risk factors for RLDH. In a review of the incidence of RLDH, the proportion of male patients was found to be 64%, but no research assessing RLDH by sex was identified [12]. Swartz and Trost [13] showed in their study that RLDH was not influenced by sex or age. As a consequence of these inconsistent results, a meta-analysis determined that age, sex, lumbar herniation level, and side of herniation were not significant risk factors for RLDH [14]. In our study, most of the patients who underwent interventional procedures for RLDH were women over the age of 50. The majority of procedures were applied at the L4–5 and L5-S1 level and on the left side.

A meta-analysis of the effectiveness of anticonvulsants in the treatment of low back and lumbar radicular pain found moderate- to high-quality evidence that anticonvulsants are ineffective in LRP, and gabapentinoids are associated with a high risk of adverse effects [15]. One review found that duloxetine was moderately effective in chronic low back pain, while nonsteroidal anti-inflammatory drugs (NSAIDs) were less effective compared to previous reviews [16]. In another review of spine-related pain in older people, low-dose opioids were recommended if treatment with low-dose gabapentin, pregabalin, duloxetine, and other drugs is ineffective [17]. In the present study, we found that 54.3% of patients with chronic low back pain used gabapentin or pregabalin, 15.7% used duloxetine, 12.2% used opioids, and 7% used NSAIDs. During the follow-up period, patients’ medication use was not altered, and it was observed that the use of medication had no effect on the success of the procedure. This may be because the medications used by the patients, the steroid administered during the procedure, and radiofrequency therapy have different mechanisms of action.

In a prospective evaluation of risk factors in RLDH, it was determined that previous surgical and intraoperative factors may play an important role [18]. In a review investigating the correlation between discectomy size and the use of instrumented fusion with recurrent herniation, it was concluded that there was conflicting data regarding which factors increase the risk of recurrent herniation [19]. In our study, 54.4% of the patients had undergone surgery at least once, and stabilization was not observed on MRI in 52.6% of patients. There was no significant difference in NRS scores during the 6-month follow-up period between patients with and without stabilization on MRI. This suggests that stabilizations do not affect the success of the procedure. Comprehensive studies evaluating the details of previous lumbar surgical interventions such as the discectomy size, fusion, and the use of screws are needed to obtain more accurate data.

In their comprehensive evidence-based guidelines, the American Society of Interventional Pain Physicians (ASIPP) evaluated fluoroscopy-guided CESI, TESI, and lumbar interlaminar injections as level 1 evidence and issued a strong recommendation for long-term effectiveness [20]. In a study evaluating the efficacy of TESI to the ventral epidural region in RLDH, TESI was effective for LRP during a 6-month follow-up period, and 54% of patients did not require reoperation [6]. We did not come across the combined application of TESI and DRG PRF treatment for RLDH in the literature. However, some studies report the results of combined TESI and DRG PRF therapy in LRP associated with LDH. In a study comparing combined TESI and DRG PRF with TESI alone, the TESI and DRG PRF group demonstrated significantly greater improvement in NRS and Oswestry Disability Index (ODI) scores over 3 months compared to the TESI monotherapy group [7]. In another study, a significant reduction in pain was observed in 66.6% of patients at 3 months after combined TESI and DRG PRF therapy in LDH-related LRP [9]. Another study comparing the effectiveness of combined TESI and DRG PRF with TESI alone found that combined treatment resulted in significantly greater improvement in VAS scores during the first 3 months compared to TESI alone, but this beneficial effect disappeared by the 4th month [8]. Significant enhancements in VAS and ODI scores were observed in a comparative study that evaluated the 4-month outcomes of DRG PRF treatment alone in chronic LRP management [21]. In the current study, to enhance the effectiveness of TESI alone, patients in both groups received the combination of TESI and DRG PRF. At 6 months post-procedure, 74% of patients in the TESI and DRG PRF group had significantly reduced pain due to RLDH. The use of DRG PRF in conjunction with TESI may provide more pain relief than TESI alone in RLDH. Larger and more comprehensive studies investigating the effectiveness of DRG PRF therapy in RLDH may yield more accurate data.

In a prospective study comparing the efficacy of CESI and TESI in RLDH, both groups showed similar results in terms of patient function and pain 6 months after injection [5]. In another retrospective study that assessed the effects of TESI alone and TESI combined with CESI in RLDH, the group that received TESI and CESI exhibited a statistically significant improvement in functional capacity and pain for 6 months [4]. We were unable to find any literature that addressed CESI for the management of RLDH with TESI and DRG PRF. In our study, the group with adjuvant CESI with TESI and DRG PRF treatment showed a greater percentage of patients who benefited from treatment only in the first month after the procedure compared to the other group. Our findings indicate that the addition of CESI to DRG PRF and TESI made no difference in the success of treatment for LRP associated with RLDH. This suggests that patients have sufficient pain relief over 6 months with TESI and DRG PRF therapy, without the need for additional steroid administration via the CESI procedure.

This study has some limitations. One of these is the retrospective design of the study and the limited number of patients. In addition, the fact that the details of the LDH operations performed by the patients could not be determined is another limitation. In this study, both groups received DRG PRF treatment in combination with TESI. Studies including patient groups in which TESI and DRG PRF treatments alone are administered may provide a clearer evaluation of the efficacy of combination therapy. Furthermore, the presence of other chronic painful diseases such as migraine and fibromyalgia was not reported in this study. In order to determine the efficacy of treatment in RLDH more clearly, it may be beneficial to exclude such chronic painful diseases in future studies. Moreover, because of the retrospective design, we could not evaluate the functional status or quality of life of the patients. Prospectively designed studies with a larger number of patients, longer follow-up protocols, assessments of quality of life and functional status, and detailed records of previous LDH surgeries will provide clearer insight on the factors impacting treatment success.

## 5. Conclusions

TESI and DRG PRF therapy were effective in treating LRP associated with RLDH over a 6-month follow-up period, and the addition of CESI did not improve treatment success. Therefore, we conclude that TESI and DRG PRF therapy can provide adequate pain palliation for 6 months in RLDH-related LRP. Additional steroid administration through CESI may not be necessary. In addition to decreasing the number of interventions performed, reducing the steroid dose in this way may help prevent many possible steroid-related side effects such as infection, hypertension, fluid retention, edema, weight gain, and hormonal changes.

## Figures and Tables

**Table 1:** Sociodemographic and clinical features of the patients.

		TESI + DRG PRF (*n* = 27)	TESI + DRG PRF + CESI (*n* = 30)	*p* Value
**Sex**	**Female**	21 (77.8%)	22 (73.3%)	
	**Male**	6 (22.2%)	8 (26.7%)	0.7 ^a^
**Age (years)**		56.5 ± 13.9	58.8 ± 12.3	0.5 ^b^
**Symptom duration (months)**		6 (3–60)	6 (3–72)	0.5 ^d^
**Injection side**	**Right**	9 (33.3%)	7 (23.3%)	0.6 ^c^
	**Left**	16 (59.3%)	19 (63.4%)	
	**Bilateral**	2 (7.4%)	4 (13.3%)	
**Injection level**	**L4–5**	4 (14.8%)	8 (26.7%)	0.5 ^c^
	**L5-S1**	4 (14.8%)	4 (13.3%)	
	**L4–5 and L5-S1**	19 (70.4%)	18 (60%)	
**Number of LDH surgeries**	**1**	14 (51.9%)	17 (56.7%)	0.9 ^c^
	**2**	11 (40.7%)	11 (36.7%)	
	**≥3**	2 (7.4%)	2 (6.6%)	
**MRI findings**	**No stabilization**	15 (55.6%)	15 (50%)	0.7 ^a^
	**Stabilization**	12 (44.4%)	15 (50%)	
**Medical treatment**	**Gabapentinoids**	17 (68%)	14 (73.7%)	0.7 ^a^
	**Duloxetine**	6 (24%)	3 (15.8%)	0.7 ^e^
	**Opioids**	3 (12%)	4 (21.1%)	0.7 ^e^
	**NSAID**	2 (8%)	2 (10.5%)	>0.9 ^e^
**NRS Score**	**Pretreatment**	7 (6–8) (7 ± 0.7)	7 (6–8) (7.3 ± 0.6)	0.1 ^d^
	**Posttreatment 1 month**	3 (0–7) (3.2 ± 1.6)	3 (0–7) (2.9 ± 2)	0.5 ^d^
	**Posttreatment 3 months**	3 (0–7) (3.4 ± 1.8)	3 (0–7) (2.9 ± 2.2)	0.4 ^d^
	**Posttreatment 6 months**	3 (0–7) (3.4 ± 1.8)	3 (0–7) (3.1 ± 2.2)	0.7 ^d^

Values are presented as *n* (%), mean ± standard deviation, and median (minimum–maximum). ^a^ Chi-square test, ^b^ independent-samples *t* test, ^c^ Fisher–Freeman–Halton test, ^d^ Mann–Whitney U test, ^e^ Fisher’s exact test, TESI: transforaminal epidural steroid injection, DRG PRF: dorsal root ganglion pulsed radiofrequency, CESI: caudal epidural steroid injection, NSAID: nonsteroidal anti-inflammatory drugs, NRS: Numeric Rating Scale, MRI: magnetic resonance imaging.

**Table 2:** Sociodemographic and clinical features of patients with successful treatment *.

		TESI+ DRG PRF (*n* = 20)	TESI+ DRG PRF +CESI (*n* = 21)	*p* Value
**Age (years)**		56.3 ± 13.3	58.2 ± 12.9	0.6 ^b^
**Sex**	**Female**	17 (85%)	15 (71.4%)	0.5 ^e^
	**Male**	3 (15%)	6 (28.6%)	
**Symptom duration (months)**		16 (3–60)	6 (3–72)	0.2 ^d^
**Injection side**	**Right**	6 (30%)	5 (23.8%)	0.9 ^c^
	**Left**	12 (60%)	14 (66.7%)	
	**Bilateral**	2 (10%)	2 (9.5%)	
**Injection level**	**L4–5**	4 (20%)	8 (38.1%)	0.6 ^c^
	**L5-S1**	1 (5%)	1 (4.8%)	
	**L4–5 and L5-S1**	15 (75%)	12 (57.1%)	
**Number of LDH surgeries**	**1**	12 (60%)	12 (57.1%)	>0.9 ^c^
	**2**	7 (35%)	8 (38.1%)	
	**≥3**	1 (5%)	1 (4.8%)	
**MRI**	**No stabilization**	13 (65%)	14 (66.7%)	>0.9 ^c^
	**Stabilization**	7 (35%)	7 (33.3%)	
**Medical Treatment**	**Gabapentinoids**	11 (61.1%)	7 (63.6%)	>0.9 ^e^
	**Duloxetine**	5 (27.8%)	1 (9.1%)	0.6 ^e^
	**Opioids**	1 (5.9%)	2 (18.2%)	0.5 ^e^
	**NSAID**	2 (11.1%)	2 (18.2%)	0.6 ^e^
**NRS**	**Pretreatment**	7 (6–8) (7.3 ± 0.6)	8 (6–8) (7.5 ± 0.6)	0.2 ^d^
	**Posttreatment 1 month**	3 (0–4) (2.6 ± 1.2)	2 (0–4) (2.1 ± 1.5)	0.3 ^d^
	**Posttreatment 3 months**	3 (0–4) (2.6 ± 1.2)	2 (0–4) (2 ± 1.5)	0.2 ^d^
	**Posttreatment 6 months**	3 (0–4) (2.6 ± 1.2)	2 (0–4) (2 ± 1.5)	0.3 ^d^

* Defined ≥ 50% or 4-point decrease in NRS score at posttreatment 6 months compared to pretreatment score. Values are presented as *n* (%), mean ± standard deviation, and median (minimum–maximum). ^b^ Independent-samples *t* test, ^c^ Fisher–Freeman–Halton test, ^d^ Mann–Whitney U test, ^e^ Fisher’s exact test. TESI: transforaminal epidural steroid injection, DRG PRF: dorsal root ganglion pulsed radiofrequency, CESI: cauda epidural steroid injection, NSAID: nonsteroidal anti-inflammatory drugs, NRS: Numeric Rating Scale, MRI: magnetic resonance imaging. NRS scores at 1, 3, and 6 months after the procedure did not differ statistically according to the number of previous LDH surgeries.

**Table 3:** Comparison of NRS values according to findings of stabilization on MRI.

		No Stabilization (*n* = 30)			Stabilization (*n* = 27)		
		TESI+ DRG PRF (*n* = 15)	TESI + DRG PRF + CESI (*n* = 15)	*p* Value	TESI+ DRG PRF (*n* = 12)	TESI+ DRG PRF + CESI (*n* = 15)	*p* Value
**NRS**	**Pretreatment**	7 (6–8)	7 (6–8)	0.7 ^d^	7 (6–8)	7 (6–8)	0.6 ^d^
	**Posttreatment 1 month**	3 (0–7)	2 (0–5)	0.2 ^d^	7.3 ± 0.6	3.7 ± 1.9	0.8 ^b^
	**Posttreatment 3 month**	3 (0–7)	2 (0–5)	0.1 ^d^	3.8 ± 1.9	4.1 ± 2	0.8 ^b^
	**Posttreatment 6 month**	3 (0–7)	2 (0–5)	0.2 ^d^	3.8 ± 1.9	4.3 ± 1.9	0.5 ^b^

Values are presented as *n* (%), mean ± standard deviation, and median (minimum–maximum). ^b^ Independent-samples *t* test, ^d^ Mann–Whitney U test. TESI: transforaminal epidural steroid injection, DRG PRF: dorsal root ganglion pulsed radiofrequency, CESI: cauda epidural steroid injection, NSAID: nonsteroidal anti-inflammatory drugs, NRS: Numeric Rating Scale, MRI: magnetic resonance imaging.

**Table 4:** Comparison of NRS values according to use of medical treatment for pain.

	Used Pain Medication (*n* = 44)			No Pain Medication (*n* = 13)		
NRS	TESI + DRG PRF (*n* = 25)	TESI + DRG PRF + CESI (*n* = 19)	*p* Value	TESI + DRG PRF (*n* = 2) *	TESI + DRG PRF + CESI (*n* = 11)	*p* Value
**Pretreatment**	7 (6–8)	7 (6–8)	0.2 ^d^		8 (6–8)	
**Posttreatment 1 month**	3.2 ± 1.6	3.4 ± 1.9	0.8 ^b^		1.9 ± 1.9	
**Posttreatment 3 month**	3.5 ± 1.7	3.6 ± 2.1	0.9 ^b^		1.9 ± 1.9	
**Posttreatment 6 month**	3.5 ± 1.7	3.8 ± 2	0.5 ^b^		1.9 ± 1.9	

Values are presented as *n* (%), mean ± standard deviation, and median (minimum–maximum). ^b^ Independent-samples *t*-test, ^d^ Mann–Whitney U test. * Not included in the statistical analysis due to insufficient amount of data. TESI: transforaminal epidural steroid injection, DRG PRF: dorsal root ganglion pulsed radiofrequency, CESI: cauda epidural steroid injection.

## Data Availability

The data supporting the findings of this study have been uploaded to the journal system and are available upon reasonable request from the corresponding author.
